# Epigenetic Modifications Induced by Nutrients in Early Life Phases: Gender Differences in Metabolic Alteration in Adulthood

**DOI:** 10.3389/fgene.2019.00795

**Published:** 2019-09-11

**Authors:** Emanuela A. Greco, Andrea Lenzi, Silvia Migliaccio, Sandra Gessani

**Affiliations:** ^1^Section of Medical Pathophysiology, Endocrinology and Food Sciences, Department of Experimental Medicine, Sapienza University of Rome, Rome, Italy; ^2^Department of Movement, Human and Health Sciences, Foro Italico University of Rome, Rome, Italy; ^3^Center for Gender-Specific Medicine, Istituto Superiore di Sanità, Rome, Italy

**Keywords:** epigenetics, nutrition, endocrine-disrupting chemicals, development, gender

## Abstract

Metabolic chronic diseases, also named noncommunicable diseases (NCDs), are considered multifactorial pathologies, which are dramatically increased during the last decades. Noncommunicable diseases such as cardiovascular diseases, obesity, diabetes mellitus, cancers, and chronic respiratory diseases markedly increase morbidity, mortality, and socioeconomic costs. Moreover, NCDs induce several and complex clinical manifestations that lead to a gradual deterioration of health status and quality of life of affected individuals. Multiple factors are involved in the development and progression of these diseases such as sedentary behavior, smoking, pollution, and unhealthy diet. Indeed, nutrition has a pivotal role in maintaining health, and dietary imbalances represent major determinants favoring chronic diseases through metabolic homeostasis alterations. In particular, it appears that specific nutrients and adequate nutrition are important in all periods of life, but they are essential during specific times in early life such as prenatal and postnatal phases. Indeed, epidemiologic and experimental studies report the deleterious effects of an incorrect nutrition on health status several decades later in life. During the last decade, a growing interest on the possible role of epigenetic mechanisms as link between nutritional imbalances and NCDs development has been observed. Finally, because of the pivotal role of the hormones in fat, carbohydrate, and protein metabolism regulation throughout life, it is expected that any hormonal modification of these processes can imbalance metabolism and fat storage. Therefore, a particular interest to several chemicals able to act as endocrine disruptors has been recently developed. In this review, we will provide an overview and discuss the epigenetic role of some specific nutrients and chemicals in the modulation of physiological and pathological mechanisms.

## Introduction

A significant increase in human longevity has been observed in the last two decades, and life expectancy exceeds the age of 80 years in several countries (World Health Organization (WHO)) with a proportional increase of chronic diseases (Figueira et al., 2016). Noncommunicable diseases (NCDs), such as diabetes, sarcopenia, osteoporosis, cardiovascular diseases, neurological disorders, and cancers, increase with age and seriously affect both subject’s life and healthcare systems (Troesch et al., 2015). In fact, NCDs induce several and complex clinical manifestations that lead to a gradual deterioration of health status and quality of life of affected individual, making the subject frail and at greater risk of disability and mortality. Then, supporting healthy aging by preventing NCDs is a major priority for agencies such as the World Health Organization (WHO) and the United Nations [World Health Organization (WHO), 2018]. In particular, WHO has identified unhealthy diets, sedentary behaviors, excessive alcohol consumption, tobacco use, and pollution among the main modifiable risk factors, with nutrition as an important determinant of human health throughout life (Eggersdorfer and Walter, 2011).

Nutrition has a pivotal role in maintaining health, and dietary imbalances represent major determinants favoring chronic diseases through metabolic homeostasis alterations. Adequate nutrition and specific nutrients are important in all periods of life, but they appear essential during specific times such as in utero life and early years of postnatal life. In this context, large amount of epidemiologic and experimental data show that imbalanced diet can induce health consequences several decades after exposure, and during the last decade, an increased interest has been observed on the possible role of epigenetic mechanisms as link between nutritional imbalances and NCD development (Block and El-Osta, 2017).

Interestingly, the “developmental origins of adult disease” hypothesis originated in 1989 from epidemiological studies by David Barker and colleagues (Barker et al., 1989a; Barker et al., 1989b) that showed newborns with small weight at birth were at a major risk of heart failure in later phases of life. Hales and Barker (Hales and Barker, 1992) used the term “programming” to describe the “permanent or long-term change in the structure or function of an organism resulting from a stimulus or insult acting at a critical period of early life.” Afterward, the concept of epigenetics was introduced to support the programming theory. Epigenetics can be described as cell-specific reversible modifications in DNA chromatin structure that modulate gene expression without altering DNA sequence. Epigenetic factors are heritable from cell to daughter cell within the same organism, and there is growing evidence that this heritability can be transgenerational among organisms (Heard and Martienssen, 2014; van Otterdijk and Michels, 2016). Indeed, the genetic heritage of each living being contains both DNA sequence information and epigenetic information, and their interaction maintains the function of organs and cells. The most studied epigenetic modifications are DNA methylation, histone modification, chromatin remodeling, and noncoding RNA, which all require the involvement of transcription factors. Further, during the last decades, several studies have confirmed the existence of specific human genes able to confer different susceptibilities to diseases (Jirtle and Skinner, 2007).

## Epigenetics Alteration Upon Early Exposure to Altered Diet and Metabolic Conditions

Transgenerational effects on metabolism and metabolic diseases have been known and studied before the advent of the field of epigenetics. In fact, the evaluation and characterization of children born during the Dutch Winter Famine (Lumey et al., 1993) showed a link between maternal nutrition and risk of metabolic disorders later in life, such as a Swedish study, which found that paternal and grand-paternal nutrition during childhood increased mortality for cardiovascular diseases and diabetes in later decades of life (Kaati et al., 2002).

These studies and following epidemiological observations show that unhealthy nutrition, not only undernutrition but also overnutrition, during in utero and early postnatal life increases susceptibility to metabolic alterations later in life by acting during the critical period of growth and by probably causing a mismatch between early and adult nutritional environments.

Maternal obesity is increasing, and several human and animal studies have demonstrated that offspring of obese mothers or mothers exposed to a high-fat diet present increased weight and fat mass at birth and during growth have an increased risk of developing nonalcoholic fatty liver disease (NAFLD), insulin resistance, altered glucose tolerance, obesity, hyperphagia, hypertension, and cardiovascular damage (Laker et al., 2013). Also, obesity and high-fat diet are associated with elevated circulating lipids that cross the placenta, through specific fatty acid transporters amplified in obese pregnant women, and they modulate cell signaling pathways by acting as ligands for nuclear receptors and altering gene expression by DNA hypermethylation. It seems that maternal high lipid levels interfere with the hypothalamic expression of leptin receptor, pro-opiomelanocortin (POMC), and neuropeptide Y in offspring, such as with the expression of SIRT1, specific factor involved in fat and glucose metabolism (Chen et al., 2008; Kim and Um, 2008). In particular, SIRT1 is principally involved in obesity, liver lipid metabolism (NAFLD), and brain neuronal degeneration. SIRT1 is a Nicotinamide Adenine Dinucleotide (NAD)+-dependent protein deacetylase, and it is involved in the deacetylation of the nuclear receptors, playing a critical role in insulin resistance development (Martins, 2013). In fact, SIRT1 is involved in metabolic regulation and in the repair of DNA damage with epigenetic alterations and maintains the DNA to prevent gene modification of various genes including CYP 450 enzymes and allows rapid metabolism of xenobiotics that enter the organism. Diet changes, as observed in underdeveloped countries’ urbanization and Western countries, involve SIRT1 dysregulation, causing several alterations in transcriptional regulators and modification of chromatin that contribute to endocrine abnormalities such as insulin resistance, NAFLD, and energy balance disorders (Martins, 2017a; Martins, 2017b). Moreover, SIRT1 has been recently identified as an antiaging gene, both in humans and other animal species. SIRT1 is involved in telomere maintenance and DNA repair with its critical involvement chromosome stability and cell proliferation and is important to the regulation of other antiaging genes such as klotho, p66shc, and Forkhead box protein O1 with relevance to age-related diseases (Martins, 2018).

**Figure 1:**
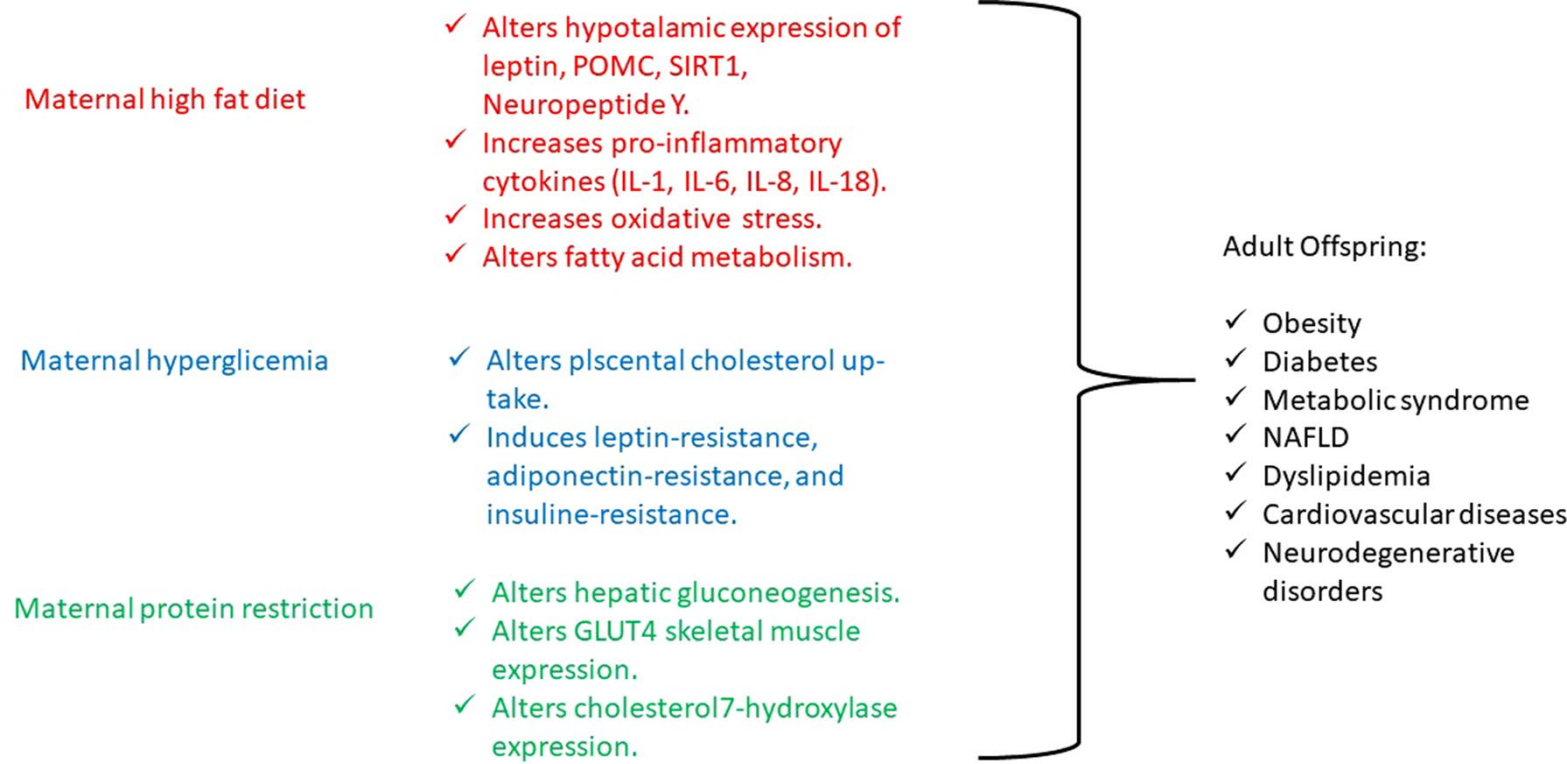
Principal biological mechanisms of diet-induced epigenetic alterations.

On the other hand, maternal elevated circulating lipids determine the activation of the inflammatory signaling, which lead to an increase in proinflammatory cytokines [tumor necrosis factor α, interleukin 1 (IL-1), IL-6, IL-8, and IL-18] and oxidative stress within the placenta, which results in an altered intrauterine and postnatal development (Laker et al., 2013). Just a few studies have investigated the effects of maternal inflammation on offspring’s postnatal life and have shown that it impairs nervous system and musculoskeletal development, while it promotes adipogenesis (Jonakait, 2007; Bayol et al., 2008; Tong et al., 2009). Interestingly, the effect of interpregnancy weight loss was studied, and a reduced risk to develop obesity and cardiovascular diseases was observed in siblings born after maternal bariatric surgery compared with those born before (Gue´nard et al., 2013). Moreover, in a mouse model, after a long-term high-fat diet, the development of obesity and mild glucose intolerance through specific gene expression alterations has been demonstrated. In particular, the authors have identified a histone acetylation among the gene expression profile in pancreatic islets, causing a dysregulation in fatty acid metabolism through the suppression of specific genes (NRF1, GABPA, MEF2A) involved in fatty acid signaling (Nammo et al., 2018). In another study conducted on pregnant rats, maternal dyslipidemia induced by an unsaturated fatty acid diet determines DNA methylation and histone acetylation in placenta and fetal liver with a subsequent accumulation of lipids in the fetal liver (Ramaiyan and Talahalli, 2018). On the other hand, the use of a hypolipidemic agent, such as *Quercus acutissima* fruit ethanol extract, exhibits antiobesity effects through inhibition of acetylation in 3T3-L1 preadipocytes and high-fat diet–fed obese mice (Hawang et al., 2017) (**Figure1**). Furthermore, recent investigations have also demonstrated that either a maternal fat overload diet or high-calorie diet can induce mitochondrial dysfunction, inflammation, and senescence-like characteristics in brown adipose cells likely leading to metabolic imbalance and increased risk of developing obesity in later phases of life (Lettieri Barbato et al., 2015; Lettieri-Barbato et al., 2017).

Like maternal obesity, gestational diabetes also has detrimental effects on both mother and fetus. Offspring of mothers with gestational diabetes present increased birth weight, adiposity, neonatal hypoglycemia, and obesity and have an increased risk of developing metabolic syndrome and type 2 diabetes later in life (Catalano, 2010). To date, several genes have been associated to diabetes, which, however, explain only a small proportion of heritability, whereas environmental factors seem to influence its pathogenesis in a significant manner. Then, the gestational diabetes represents an interesting model to study the epigenetic modifications determined by environmental influence (Nolan et al., 2011). Indeed, gestational diabetes is more frequent in daughters of diabetic mothers than in those of diabetic fathers (Harder et al., 2001; McLean et al., 2006), pointing to intrauterine glucose exposure as a relevant issue in addition to genotype (Hocher, 2014; Hocher et al., 2016; Reichetzeder et al., 2016). In fact, intrauterine exposure to hyperglycemia determines an impairment in placental cholesterol uptake and alters placental methylation of leptin and adiponectin, hormones that regulate energy balance and insulin sensitivity, leading to the development of both leptin and insulin resistance. Moreover, animal and human studies show that leptin and insulin resistance, such as undernutrition, act on hypothalamic receptors and appetite circuits leading to postnatal hyperphagia, decreased satiety, and subsequent development of metabolic syndrome (Block and El-Osta, 2017) (**Figure1**).

Finally, maternal restriction of proteins, folate, methionine, and B vitamins during periconceptional period, gestation, and lactation increases the risk of lower weight at birth and increased central adiposity, fatty liver, blood pressure dysregulation, and myocardium hypertrophy in offspring as consequences of an altered DNA methylation (Wu, 2009; Gueant et al., 2013). In particular, in an experimental mouse model, it has been demonstrated that a low-protein diet in pregnant mothers during a precocious gestational period, such as the preimplantation period, determines cardiovascular and metabolic diseases in offspring adults, through histone modifications of the *Gata6* gene (Sun et al., 2015). And, in male rat offspring, maternal protein restriction, during gestation and lactation, determines impaired glucose tolerance in adulthood by histone acetylation of the liver X receptor α, which is involved in the regulation of hepatic gluconeogenesis (Vo et al., 2013), as well as leads to histone modifications in GLUT4 promoter region in the skeletal muscle of female rat offspring (Zheng et al., 2012) and determines high cholesterol levels in adult rat offspring because of repressive changes in histone modifications at the cholesterol 7α-hydroxylase promoter (Sohi et al., 2011) (**Figure1**).

A last consideration must be made regarding alcohol consumption during pregnancy. In fact, recent animal studies show that prenatal ethanol exposure determines high fat mass at birth, altered β-cells structure, impaired glucose homeostasis, and insulin resistance (Yao and Nyomba, 2008; Dobson et al., 2012), by inducing anomaly in DNA methylation (Ungerer et al., 2013), likely due to reduced folate bioavailability and methionine levels (Halsted et al., 2002).

## Epigenetics Modifications Upon Early Exposure to Chemicals Through Food Chain

Hormones play a pivotal role through life in the regulation of fat, carbohydrate, and protein metabolism, and hormonal alterations of these processes are likely to impair metabolism and fat storage. Many natural and synthetic chemicals, found in the environment, contaminating food through food chain, possess hormonal activity. These compounds, known as endocrine disrupters (EDCs), are exogenous substances endowed with the capacity to alter the function(s) of the endocrine system and thus represent a serious risk to health both in humans and animals (International Programme for Chemical Safety) (Li et al., 2013; Maradonna and Carnevali, 2018). Endocrine-disrupting chemicals belong to a heterogeneous class of chemicals dispersed in the environment. These compounds alter many aspects of the endocrine-metabolic homeostasis because of their ability to mimic and/or antagonize the biological activity of endogenous hormones (Pande et al., 2019), likely binding to specific receptors. Although the main EDC effect is on the reproductive system (McLachlan et al., 1984), growing evidence shows that some compounds can also impair body weight regulation by affecting metabolism (Migliaccio et al., 1996) and functional activity of adipocytes, often leading to obesity. These EDCs are defined as “obesogens” (Grun and Blumberg, 2006). Several chemicals have comprised obesogens with estrogen properties, such as tributyltin (TBT), generally used as biocide in antifouling paints applied to the hulls of ships; diethylstilbestrol, used to enhance fertility in farm animals; dichlorodiphenyltrichloroethane (DDT) and its breakdown product dichlorodiphenyl-dichloroethylene, used as insecticide; bisphenol A (BPA), used in the manufacture of plastics; polybrominated diphenyl ethers and 4-nonylphenol, used for industrial proceedings; parabens, generally used as antimicrobial agents for the preservation of personal care products, foods, pharmaceutical products, and paper products; phytoestrogens, naturally produced by plants and assumed by humans *via* ingestion of edible plants (Darbre, 2015). “Interestingly, several animal and human evidence shows that the exposure to obesogens, both prior to birth in utero and during neonatal period, leads to altered body weight at birth (both high weight and low weight) and increased body weight and obesity during growth with an increase in fat cell number permanently into adult life (Janesick and Blumberg, 2011). Moreover, many studies highlight that such effects can also be inherited through future generations even in the absence of additional exposure. Transgenerational studies have revealed that TBT exposure of pregnant mice generates offspring of both genders with larger fat deposits, and this phenotype is inherited up to the third generation, even without further TBT exposure (Chamorro-Garcia et al., 2013; Janesick and Shioda, 2014). Other heritable traits toward obesity in rodents have been observed after exposure to BPA, phthalates (Manikkam et al., 2013), and DDT (Skinner et al., 2013).

Obesogens induce weight gain by increasing both the number and size of adipocytes, by altering the endocrine pathways responsible for adipose tissue development, by changing lipid homeostasis, and by promoting adipogenesis and lipid accumulation. These events might occur through multiple mechanisms, such as interference with Peroxisome Proliferator-Activated Receptors (PPARs) and steroid receptors, alteration in fat cell recruitment, shifting of appetite, satiety, and food preferences (Darbre, 2017). In particular, it is thought that early life exposure to EDCs might influence epigenetic programming of obesity *via* the capacity of these compounds to bind nuclear receptors and other transcription factors and thus to influence consequent gene expression. For example, nuclear receptors, such as steroid receptors, can directly bind hormone-response elements present in the DNA upon activation by single or multiple ligands. Furthermore, they are able to recruit chromatin-modifying complexes including methyltransferases and acetyltransferases, which directly alter epigenetic marks involved in the regulation of target genes (Ozgyin et al., 2015). Therefore, EDCs can change the local chromatin state as well as modulate the expression of DNA or histone methyltransferases by activating or inhibiting nuclear receptors and other transcription factors (Rissman and Adli, 2014).

Among EDCs, phytoestrogens represent a diverse group of natural chemicals with structural and functional similarities to endogenously produced mammalian estrogens, able to bind the nuclear receptors and thus endowed of significant estrogen receptor (ER) modulatory activities. They are present in fruits, vegetables, and whole grains commonly consumed by humans, as well as in many dietary supplements, and are widely marketed as natural alternatives to estrogen replacement therapy. Recently, the nutritional changes leading to the inclusion of soy-derived products into human diets have consistently enhanced the exposure to these compounds. In fact, soy products are nowadays important components of food products consumed in both adult and infant human diets (McCarver G et al., 2011), with variable amounts assumed in different world regions (Jefferson et al., 2009). Phytoestrogens are polyphenolic structures classified as flavonoids (or isoflavones), coumestans, lignans, and stilbenes, with isoflavones representing major compounds in dietary sources (Rietjens et al., 2017). Various beneficial health effects have been ascribed to these compounds including cardiovascular diseases, obesity, metabolic syndrome, and type 2 diabetes, as well as brain function disorders and some types of cancer (Guerrero-Bosagna and Skinner, 2014).

Phytoestrogens exert their potential health effects by different ways. Although the main mode of action relies on their binding to ER, several other pathways such as rapid nongenomic cellular response, antioxidant action, tyrosine kinase inhibition, PPAR-mediated action, and binding to nonclassic ER gp130 or aryl hydrocarbon receptor (AHR) have been largely described (Guerrero-Bosagna and Skinner, 2014). Compelling evidence suggests that epigenetic modifications link environmental insults occurring during development to disease susceptibility in the adult life. In this regard, the capacity of phytoestrogens to induce epigenetic effects has been described, in particular for the soy isoflavone genistein and to a lesser extent for daidzein and its microbial metabolite equol (Guerrero-Bosagna et al., 2008; Remely et al., 2015). A direct epigenetic effect of isoflavones was initially demonstrated upon exposure of newborn mice to coumestrol and equol that lead to increased methylation and subsequent inhibition of the proto-oncogene H-ras (Lyn-Cook et al., 1995) in both male and female mice. Furthermore, consumption of genistein was reported to alter DNA methylation pathways in mice (Day et al., 2002). In addition to direct effects, evidence has been achieved on the capacity of phytoestrogens to affect offspring methylation patterns as a result of maternal exposure. In this regard, dietary supplementation of pregnant mice with genistein altered coat color and protected A^vy^ mouse offspring from obesity development by modifying the epigenome of the fetus (Dolinoy et al., 2006).

Several studies have been carried out to assess the obesity-promoting or obesity-protective effect of maternal supplementation with phytoestrogens on the offspring. The results achieved are often difficult to compare because of several variables including the animal model, interspecies differences in isoflavone metabolism, diet composition, phytoestrogen concentration, and length of treatment, as well as confounding factors such as age and gender (Ørgaard and Jensen, 2008). In this regard, it is of interest that sex differences in human amniotic fluid levels of daidzein and genistein, with significantly higher concentrations among the female fetuses, have been reported (Jarrel et al., 2012). Likewise, genistein pharmacokinetics are faster in male rather than female rats (Sikker et al., 2001). Studies assessing the effects of in utero exposure to phytoestrogens in different preparations (genistein as supplement to standard diet, isoflavone-rich diet, soy protein–based diet) and for different periods after birth yielded contrasting results. Whereas some studies reported that in utero exposure results in a lower weight at birth (Cederroth et al., 2007; Guerrero-Bosagna and Skinner, 2009; Zhang et al., 2015), others reported obesity-promoting properties such as increased body weight and food intake (Vafeiadi et al., 2015; Jahan-Mihan et al., 2011; Jahan-Mihan et al., 2012; Cao et al., 2015; Walley and Roepke, 2018). Likewise, more than a decade ago, Ruhlen and colleagues (Ruhlen et al., 2008) reported either decreased or increased body weight of offspring upon in utero exposure depending on the period of life (adulthood vs. at birth).

Predisposition to diet-induced obesity as a consequence of prenatal following prenatal nutrient restriction has been reported to be gender related, with males more affected than females, as well as age at pubertal development (girls earlier than boys) (Rubin et al., 2017; Guerrero-Bosagna et al., 2008). Regardless of the effect induced in the offspring by in utero exposure to phytoestrogens, they appeared to be stronger in the male progeny with respect to females.

## Conclusions

Obesity and NCDs are increasing burning health problems that greatly affect worldwide population. Several factors are claimed to play a role in the development and persistence of these metabolic chronic disorders through life, such as altered diet and sedentary life. Interestingly, in the last decades, several studies have pointed out the importance of perturbance during the early phases of life in the increased number of metabolic chronic disorders. In particular, altered diet and exposure to specific chemicals through food chain appear to play a pivotal role. Further studies, however, are needed to fully characterize and confirm this hypothesis in order to apply preventive actions to successfully approach this global health problem.

## Author Contributions

All authors have equally contributed in writing, revising, and finalizing the manuscript.

## Conflict of Interest Statement

The authors declare that the research was conducted in the absence of any commercial or financial relationships that could be construed as a potential conflict of interest.
